# Study of amphipathic metabolites in cardiac pathophysiology: Insights gained from long‐chain acylcarnitines and calcium handling

**DOI:** 10.1111/jcmm.17949

**Published:** 2023-09-08

**Authors:** Hamish M. Aitken‐Buck, Ei Phyo Khaing, Regis R. Lamberts, Peter P. Jones

**Affiliations:** ^1^ Department of Physiology, HeartOtago, School of Biomedical Sciences University of Otago Dunedin New Zealand

**Keywords:** acylcarnitines, calcium, membrane perturbation, ryanodine receptor, spontaneous calcium release

Metabolomics has identified many metabolite associations with cardiovascular disease. In particular, the fat metabolism intermediates, long‐chain acyl carnitines (LCACs), have been associated with heart failure^1^ and cardiac arrhythmias.^2^ These findings have awoken a long‐dormant field of research that established LCACs as disruptors of cardiac contractility and electrophysiology.^3^ With the evolution of cardiovascular metabolomics, this preclinical research field will continue to emerge, allowing LCACs to be like ‘old actors auditioning for new roles’ in cardiac pathophysiology, as coined by McCoin and colleagues in their recent review.^4^ With this new wave of research, it is vital that we learn from methodological limitations that previously stalled the field so that new discoveries have improved validity and translatability.

LCACs have been reported to affect many aspects of cardiac physiology.^3^ However, recent research efforts have been focussed on LCAC effects on the cardiomyocyte intracellular Ca^2+^ release channel, the ryanodine receptor (RyR2).^3^ In mouse cardiomyocytes, LCAC 16:0 (palmitoylcarnitine) has been found to promote RyR2‐mediated Ca^2+^ sparks,^5^ while we have shown that LCACs 16:0 or 18:1 (oleoylcarnitine) promote spontaneous Ca^2+^ release (SCR) in HEK293 cells expressing RyR2.^6^ Interestingly, our research, which aligns with earlier reports,^7^ also found that LCACs cause intracellular Ca^2+^ accumulation, independently of Ca^2+^ released by RyR2.^6^ It is known that RyR2 activation is sensitive to the cytosolic Ca^2+^ level,^8^ including in our HEK293 cell model.^9^ Therefore, it is possible that the effect of LCACs on RyR2 and SCR might be due to an increased cytosolic Ca^2+^ presence rather than a direct effect on RyR2.

Properly addressing this mechanism requires research that accounts for factors that have long limited the translational impact of the field.^3^ From our perspective, this includes considering the membrane perturbing effects of the LCAC amphipathic structure and the translatability of extracellular LCAC concentrations used in laboratory‐based studies. For this short communication, we have used LCAC effects on RyR2‐mediated SCR as an example system to highlight how these limitations impact findings, with the hope that future research into LCACs or other amphipathic compounds will appropriately consider them.

To do this, we have exposed RyR2‐expressing HEK293 cells to LCACs 16:0 and 18:1 at concentration ranges spanning physiological (0.1 μM) and pathological (1.0 μM) levels,^10^ and an excessive (10 μM) level analogous to those commonly used in LCAC research.^3^ Circulating LCACs can reach levels in ≥50 μM^4^; however, these levels are found only in rare fatty acid oxidation disorders.

Compared to vehicle controls or the short‐chain acylcarnitine C3 (propionylcarnitine), LCACs 16:0 and 18:1 acutely increased SCR propensity and the average SCR frequency in our cell model (Figure 1A‐C). This was evident at all LCAC concentrations. At each concentration, the effect of LCAC 18:1 on SCR propensity was greater than the effect of LCAC 16:0, indicating a dependence on the fatty acyl chain length. Conversely, the effect on SCR frequency was not dependent on the LCAC fatty acyl chain length, except at the excessive concentration, where the effect of LCAC 18:1 was reduced relative to 16:0 (Figure 1C).

**FIGURE 1:**
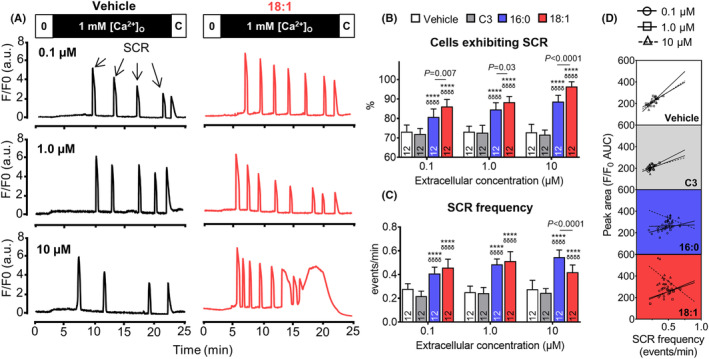
Effects of long‐chain acylcarnitines (LCACs) on spontaneous Ca^2+^ release (SCR). LCACs 16:0 and 18:1 were tested at a range of concentrations in human embryonic kidney 293 (HEK293) cells with tetracycline‐inducible expression of the cardiac ryanodine receptor (RyR2). Equivalent volume vehicle or equivalent concentration short‐chain C3 acylcarnitine were used as comparisons. (A) HEK293 cells grown on glass coverslips were induced to express RyR2 (16–20 h prior to experiment), then loaded with Fluo‐4‐AM (2 μM) for 10 min and superfused with Krebs‐Ringer Henseleit (KRH) solution containing 0–1 mM Ca^2+^ (to create 0–1 mM extracellular Ca^2+^ concentrations ([Ca^2+^]_O_)). Exposure to extracellular Ca^2+^ promoted SCR events, as depicted by arrows. Cells were superfused with caffeine (C, 20 mM) at the end of each experiment to deplete the intracellular store. Each condition was applied to cells at the beginning of each experiment. Only vehicle and LCAC 18:1‐treated cells are shown for clarity. The percentage of cells exhibiting ≥1 SCR event and the average number of SCR events under each condition shown in (B) and (C), respectively. (D) Associations between SCR frequency and fluorescence peak area. Simple linear regression lines fitted to illustrate trends. *N*‐values as indicated. All analyses performed using two‐way anova with Tukey's multiple comparisons for significant factors. ^****^ *p* < 0.0001 versus vehicle of same condition; ^δδδδ^ *p* < 0.0001 versus C3 of same concentration.

What might explain the seemingly contradictory latter finding? It is well established that LCACs can partition into phospholipid bilayers in a fatty acyl chain length dependent manner.^11^ Importantly, LCAC membrane insertion increases the membrane surface pressure and can disrupt membrane integrity.^12^, ^13^ From our SCR assay, an increase in the number of SCR events normally translates to an increase in cumulative Ca^2+^ fluorescence (‘peak area’) (Figure 1D). Note the regression lines fit in Figure 1D are for illustrative purposes only. This relationship is negative in cells exposed to excessive LCACs, suggesting that Ca^2+^ movement into the cytosol from the extracellular environment might mask discrete SCR events (see Figure 1A bottom‐right panel). This effect is likely related to LCAC insertion of the HEK293 cell membrane^12^; therefore, it is not surprising that greater SCR masking occurs following exposure to the longer hydrophobic component of LCAC 18:1 (Figure 1C).

To confirm that excessive LCACs promote Ca^2+^ passage from the extracellular environment, we assessed passive cytosolic Ca^2+^ influx in Fluo‐4‐AM loaded HEK293 cells not induced to express RyR2 prior to experimentation, which are unable to undergo SCR. Excessive LCAC 16:0 and 18:1 concentrations markedly increased passive Ca^2+^ influx (peak [Ca^2+^]_c_) in a fatty acyl chain length dependent manner, while the physiological and pathological concentrations did not (Figure 2A,B). To explore whether excessive LCACs allow the passage of Ca^2+^ specifically or have non‐specific detergent‐like effects on membrane permeability, we performed the same assay in HEK293 cells loaded with a Zn^2+^ indicator (FluoZin‐3‐AM) and perfused with 1 mM Zn^2+^. Consistent with the results found with Ca^2+^ entry, excessive, but not physiological or pathological, LCAC concentrations markedly increased Zn^2+^ influx proportionally to acyl chain length (Figure 2C,D).

**FIGURE 2:**
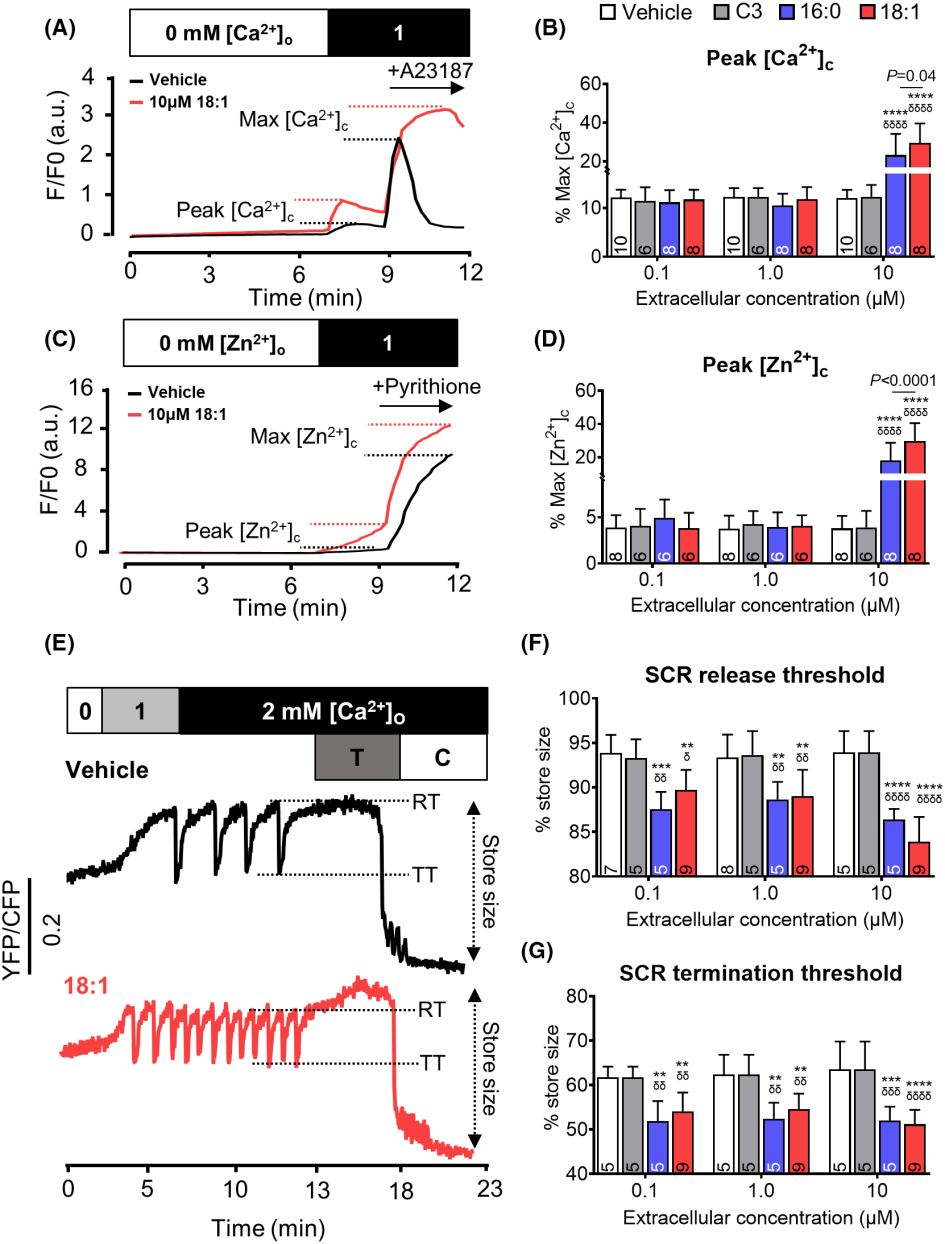
Differing effect of long‐chain acylcarnitine (LCAC) concentrations on passive ion influx and RyR2 luminal Ca^2+^ handling. LCACs 16:0 and 18:1 were tested at a range of concentrations in human embryonic kidney 293 (HEK293) cells with tetracycline‐inducible expression of the cardiac ryanodine receptor (RyR2). Equivalent volume vehicle or equivalent concentration short‐chain C3 acylcarnitine were used as comparisons. (A) HEK293 cells without RyR2 induction were loaded with Fluo‐4‐AM (2 μM, 10 min) and exposed to Krebs‐Ringer Henseleit (KRH) containing 0–1 mM Ca^2+^ ([Ca^2+^]_O_) to establish the peak cytosolic Ca^2+^ level (peak [Ca^2+^]_c_) resulting from passive Ca^2+^ influx. Cells were superfused with A23187 (10 μM, Ca^2+^ ionophore) to establish the maximal cytosolic Ca^2+^ level (Max [Ca^2+^]_c_). (B) Mean peak [Ca^2+^]_c_ levels promoted by each condition (as % of max [Ca^2+^]_c_). (C, D) Same as for (A, B), but peak cytosolic Zn^2+^ level (Peak [Zn^2+^]_c_) measured in cells loaded with FluoZin‐3‐AM (3 μM) and exposed to pyrithione (10 μM, Zn^2+^ ionophore) to establish the maximal cytosolic Zn^2+^ level (Peak [Zn^2+^]_c_). (E) HEK293 cells expressing RyR2 expression were transfected with D1ER (FRET‐based luminal Ca^2+^ sensor) 24 h prior to RyR2 induction. Cells were superfused with KRH containing 0–2 mM Ca^2+^ to promote spontaneous Ca^2+^ release (SCR) before exposure to tetracaine (T, 2 mM, RyR2 blocker) and caffeine (C 20 mM) to establish maximal and minimal luminal Ca^2+^ levels, respectively. The difference between the maximal and minimal levels was used to calculate store size. SCR onset (RT) and termination thresholds (TT) were determined from each caffeine‐responsive cell. Average threshold values (as % of store size) for each condition are shows in (F) and (G). *N*‐values as indicated. All analyses performed using two‐way anova with Tukey's multiple comparisons for significant factors versus vehicle of same volume: **p < 0.0, ^****^ *p* < 0.0001 versus C3 of same concentration: ^δ^ *p* < 0.05, ^δδ^ *p* < 0.01, ^δδδ^ *p* < 0.001, ^δδδδ^ *p* < 0.0001.

These data strongly suggest that excessive LCAC concentrations commonly used in the field to mimic disease levels exert detergent‐like effects on cell membranes, allowing non‐selective ionic movement across the membrane.^3^ This contrasts with physiological and pathological LCAC levels which promoted SCR without exerting overt membrane disruption. Therefore, to assess whether these concentrations are directly altering RyR2 function we expressed the luminal Ca^2+^ sensor, D1ER, in our cell model.^9^ LCACs had no effect on luminal Ca^2+^ store size. All LCAC concentrations did, however, significantly reduced the thresholds for RyR2‐mediated SCR onset (SCR initiated at lower store Ca^2+^ level) and SCR termination (greater Ca^2+^ store depletion required to terminate a SCR event) (Figure 2E–G). Given that physiological and pathological LCAC concentrations have these effects without changing store size or promoting overt cytosolic Ca^2+^ accumulation (Figure 2A,B), these data would be consistent with LCACs altering the luminal Ca^2+^ sensitivity of RyR2.^14^

Collectively, the outcomes of this brief communication have implications for methodology of future research of LCACs or other amphipathic metabolites. Moreover, as a by‐product of our using it as our example system, we have also gained insight into the relationship between LCACs and pro‐arrhythmic Ca^2+^ release by RyR2.

First, regarding the latter, we have learned that LCACs promote RyR2‐mediated SCR at concentrations that do not overtly affect membrane integrity. Mechanistically, this raises the question of how LCACs alter RyR2 gating. Given that LCACs alter cytosolic Ca^2+^ homeostasis,^7^ it would be logical to investigate the interaction between LCAC treatment and RyR2 phosphorylation driven by Ca^2+^/calmodulin‐dependent kinase II, which has been linked to RyR2 gating previously.^15^ Similar investigation is warranted for the effect of physiological LCAC concentrations on RyR2 carbonylation and ultrastructure arrangement, which has been shown to be altered by excessive LCAC 16:0 concentrations in mouse hearts.^5^

Second, and pertaining to the rationale for this study, we have found that excessive LCAC concentrations commonly used in the field do increase non‐specific intracellular ion influx in a manner dependent on the LCAC's amphipathic structure. This is suggestive of cell membrane disruption that could mask other physiologically relevant effects in the cell. Because of this, future research must consider the concentration of LCAC used to characterize effects in the heart or, alternatively, provide evidence that membrane integrity is maintained.

By accounting for these important aspects of LCAC methodology, the growing body of contemporary LCAC research can better identify cellular processes affected by LCAC dysregulation in cardiovascular disease. Without doing so, efforts to determine the translatability of targeting LCAC dysregulation for new therapies will lag behind the risk and prognosis associations drawn from clinical metabolomic studies.

## AUTHOR CONTRIBUTIONS

**Hamish M Aitken‐Buck:** Conceptualization (lead); data curation (equal); formal analysis (lead); funding acquisition (equal); investigation (lead); methodology (equal); project administration (equal); visualization (lead); writing – original draft (lead); writing – review and editing (lead). **Ei Phyo Khaing:** Data curation (equal); formal analysis (supporting); investigation (supporting); writing – original draft (supporting); writing – review and editing (supporting). **Regis R Lamberts:** Conceptualization (equal); data curation (supporting); methodology (equal); project administration (equal); supervision (supporting); writing – original draft (equal); writing – review and editing (equal). **Peter P Jones:** Conceptualization (equal); data curation (equal); funding acquisition (equal); methodology (equal); project administration (equal); resources (equal); software (equal); supervision (equal); writing – original draft (equal); writing – review and editing (equal).

## CONFLICT OF INTEREST STATEMENT

The authors declare that they have no conflicts of interest to disclose.

## Data Availability

The data that support the findings of this study are available from the corresponding author upon reasonable request.
